# A Treatise on Stricture of the Urethra, Containing an Account of an Improved Method of Treatment; with an Appendix Noticing the Application of a New Instrument to the Treatment of Enlarged Prostate Gland, Gleet, Fistula, and Other Diseases of the Urethra, Œsophagus, and Rectum

**Published:** 1819-10-01

**Authors:** 


*A Treatise on Stricture of the Urethra, containing an Account of an improved Method of Treatment; with an Appendix noticing the Application of a New Instrument to the Treatment of Enlarged Prostate Gland, Gleet, Fistula, and other Diseases of the Urethra, Œsophagus, and Rectum.* By JAMES ARNOTT, Member of the Royal College of Surgeons, &c. One Vol. Octavo, 183 pages, four plates. London, 1819.

WE have carefully perused this little volume, and are of opinion that it is by far the best systematic work on the subject, in the English language. It is a judicious compilation, interwoven with much original and acute observation. Into the systematic part we shall not enter, as it forms a regular elementary classic on the disease; but we shall lay before the reader some account of the new instrument invented by Mr. Arnott or his brother, together with its application to the affections stated in the title-page above.

Before describing the apparatus, however, we shall glance at two passages which arrested our attention in perusing the work; one is a hint relative to the introduction of the catheter into the bladder, the other respects the passage of a bougie through a stricture. In the *former* operation he advises the urethra to be distended with any fluid, injected through the catheter from a bag attached to its outer extremity. It is necessary, that during this operation, the penis be gently grasped, so as to prevent the return of the liquid by the sides of the instrument. "This distention of the canal, Mr. A. avers, removes every obstacle to the passing of the catheter, until it reaches the stricture itself, (an object of considerable importance where it is necessary to pass an instrument in an irritable urethra) and the orifice of the stricture being also filled and distended by this unirritating fluid, is open for the reception of the instrument's point." By this mode of distention too, a great facility is given to the extraction of stones, or of any instrument that has been broken and is retained in the canal.

In respect to the bougie, we shall introduce the following short extract from the work.

"As a bougie of wax cloth, of the small size, that will pass through a (narrow) stricture, has not strength sufficient to retain its form while passing down the urethra; as much danger of piercing the urethra attends the use of a sharp pointed metallic instrument in such a case; and as the catgut, which has been commonly substituted for these, on account of its having greater strength with less bulk than any other substance, here applicable, is liable, if retained, to swell from the moisture of the canal, so as to become of difficult extraction, and consequently to produce inflammation which may entirely close the remaining opening; I have for some time made use of the following expedient: I pass a full-sized canula down to the stricture, ascertain the situation of the opening, and then introduce through the canula the smallest wax cloth bougie. This being unimpeded by the contact of the urethra, searches for the opening with great advantage; and if the first one fail, others may be successively used, without exciting new irritation." P. 89.

We now come to the description of Mr. Arnott's improved instrument, the dilator.

"A tube of oiled silk, lined with the thin gut of some small animal to make it air tight, and then attached upon the extremity of a small canula, by which it is distended with air or water from a bag or syringe at the outer end, with a stop-cock or valve to keep the air in when received, is a description of the apparatus.

"The thinnest silk ribbon, of different breadths, with the edges neatly sewed, so as to make it tubular, and then varnished with prepared linseed oil, which dries upon it, and leaves the surface perfectly smooth and soft, is what I have found to answer best. The gut of any small animal will form the lining; but that of the cat is preferable, on account of its thinness and strength. The canula may be of elastic gum, or of the flexible metal used to make the metallic bougie, or of silver; and the injected air may be retained, either by a stop-cock at the outer extremity of the canula, or by a valve at the silk tube or bag itself. This last method is the only one applicable to the insulated dilator, which is a short length or bulb of the lined silk, to be distended and left in the canal, and having a bit of canula in the centre, for the free passage of the urine; the patient while wearing it being frequently at liberty to follow his usual occupation." P. 98.

This species of dilator may be modelled to every form or dimension. It possesses strength to bear any degree of pressure that may be useful; and this pressure can, at all times, be increased or diminished by the injection of more air, or by opening the cock. The action of the dilator is always proportioned to the extent of the dilator's surface, which is depressed by its bearing against it.

Though, in general, the dilator passes as easily down to the stricture as a small bougie, yet, on some occasions, especially in irritable urethræ, Mr. A. introduces it through a smooth canula. As soon as the bag is sufficiently within the stricture or strictures, as much air is to be injected into it as the patient can easily bear; and during the time it remains in the urethra, the future admission or escape of the air is regulated by his sensations. Much pain should never be given by the distension; "for it is wonderful how little force is required to dilate strictures, when it is divested of the irritating, and consequently counteracting quality, that often attends it when produced by the bougie."

Mr. A. states that he has sometimes widened narrow strictures, sufficiently to admit the proper silk tube dilator, by a single or double small gut, introduced upon a fine wire, projecting from the canula, distended, and the wire then extracted.

"An important advantage peculiar to the dilator, arising when it is not distended too forcibly, is, that it yields for a time to the violent spasm that is occasionally induced by passing any foreign body through a stricture. When a bougie yields, its power of distension is immediately lost; but the force of the dilator is only concentrated, and returning to the charge, ultimately overcomes every obstruction." P. 108.

In the Appendix, Mr. Arnott enumerates the other cases, besides urethral stricture, in which the dilator may be usefully employed. 1st. In strictures of the œsophagus and rectum. 2. In chronic enlargement of the prostate gland. 3d. In urinary calculus. 4th. In the second stage of gonorrhœa and gleet. In these last, our author fills with an astringent injection a *pervious* dilator bag, lying in the diseased portion of the urethra. Thus the part is kept constantly moistened with it; " and it is plain, (says Mr. A ) that every good effect that can be obtained from such a topical application will be procured, with the additional advantage that the injection is confined almost completely to the diseased part." P. 168.—He thinks too, that the mechanical support given to the morbid structure is of great and peculiar advantage in gleets and gonorrhœa, on the same principle as bandages in sores and other superficial chronic inflammations. 5th. In fistulæ.

" In those very distressing cases, where a fistulous communication exists between the bladder and rectum, the use of the dilator promises greater chance of recovery than any other expedient; if, indeed, any other exists. A large dilator passed up the rectum beyond the fistulous communication, and there retained by the canula, which supplies it with air, being secured at the anus, with an opening through it, by which the fæces may descend, and from this opening a silk tube continued, to carry the fæces out per anum, to obviate the possibility of their entering the fistula, would constitute the apparatus." P. 170.

In fistula in ano, Mr. Arnott thinks that the necessity of cutting open the sinus would often be obviated by compressing its sides closely together with the dilator introduced into the rectum, after causing some tendency to adhesive inflammation, by irritating injections.

6. In hæmorrhages from canals, as from the urethra or rectum, the dilator promises to be of considerable utility. In hæmorrhage from the nose, the dilator is easily introduced, and may prove very efficient.

Our author proposes a broad bag dilator as a good substitute for the pessary, in descent of the uterus. This we know has been tried; but some unlucky and untimely *explosions* have brought the gas pessaries into disgrace. In prolapsus ani, Mr. A. thinks the air dilator better adapted than any kind of bandage, as it supports the body of the gut, and may be worn in cases where the intestine has a tendency to protrude in the erect position of the body.

We understand, that at the last sitting of the Medico-Chirurgical Society, Mr. Astley Cooper spoke in high terms of Mr. Arnott's dilator, and alluded to an interesting case where the instrument had superseded the use of the gorget or scalpel in the removal of a stone from the bladder. The following outline of the case mentioned by Mr. Cooper, was kindly forwarded to us by a surgeon of this metropolis.

"A strong man, 60 years of age, had stone in the bladder, for which lithotomy was performed a year ago. The rectum was cut into at the time, and a fistula yet remained between it and the urethra, when he came to London, about the end of May last, with great irritation of the parts and frequent spasms of the bladder. With a view of curing the fistula, an opening was made by Mr. A. Cooper, from the perinæum to the membranous part of the urethra, through which a short catheter was passed, that the urine might flow clear of the fistula, and allow it to heal. On examining the bladder, after the general relief obtained by this step, a stone was still found in it. As an opening already existed from the perinæum, this was thought a good opportunity for trying to extract the stone, without further cutting, by widening the passage with the *dilator*. The attempt was made, with scarcely any pain to the patient, and the parts yielded so much, in less than thirty hours, that the common stone forceps was introduced as easily as after the division of the prostate by the common gorget. A stone of the size of a small walnut was then extracted, having for its nucleus a fragment that remained after the first operation. The parts collapsed immediately, and he had no consequent pain, irritation, or fever. Three hours after the removal of the stone, he voided his urine in the usual way, and the capability of retaining it increased as the irritation from the stone subsided. He continued to receive company; went into his drawing-room on the fifth day after the operation; and on the ninth rode out in a carriage, by which time the opening in the perinæum was entirely healed.

"For this case the dilator included a catheter, by which the urine passed during the continuance of the instrument in the canal."

Upon the whole, we cannot too strongly recommend this publication of Mr. Arnott's, not only as the best work on stricture generally, but as giving publicity to an instrument which promises to be of essential benefit to operative surgery.

*A Practical*

